# Strategies for integrating artificial intelligence and cognitive assessment to predict disability progression in relapsing-remitting multiple sclerosis: A model development study

**DOI:** 10.1371/journal.pdig.0001722

**Published:** 2026-09-25

**Authors:** Chao Zhu, Xin Zhang, Anneke van der Walt, Bruce Taylor, Mastura Monif, Tomas Kalincik, Jeanette Lechner-Scott, Katherine Buzzard, Trevor Kilpatrick, Michael Barnett, Zhen Zhou, Vilija Jokubaitis, Melissa Gresle, David Darby, Deval Mehta, Zongyuan Ge, Helmut Butzkueven, Daniel Merlo

**Affiliations:** 1 Department of Neuroscience, School of Translational Medicine, Monash University, Melbourne, Victoria, Australia; 2 Department of Electrical and Computer Systems Engineering, Monash University, Melbourne, Victoria, Australia; 3 AIM for Health Lab, Faculty of IT, Monash University, Melbourne, Victoria, Australia; 4 Discipline of Pediatric and Child Health, School of Clinical Medicine, University of New South Wales, Sydney, New South Wales, Australia; 5 Department of Neurology, Alfred Hospital, Melbourne, Victoria, Australia; 6 Menzies Institute for Medical Research, University of Tasmania, Hobart, Tasmania, Australia; 7 Department of Neurology, Neuroimmunology Centre, Royal Melbourne Hospital, Melbourne, Victoria, Australia; 8 CORe, Department of Medicine, University of Melbourne, Melbourne, Victoria, Australia; 9 Hunter New England Health, New Lambton, New South Wales, Australia; 10 University of Newcastle, Newcastle, New South Wales, Australia; 11 Eastern Health Clinical School, Monash University, Box Hill, Victoria, Australia; 12 Florey Department of Neuroscience and Mental Health, University of Melbourne, Melbourne, Victoria, Australia; 13 Brain and Mind Centre, University of Sydney, Sydney, New South Wales, Australia; 14 Sydney Neuroimaging Analysis Centre, Camperdown, New South Wales, Australia; 15 School of Public Health and Preventive Medicine, Monash University, Melbourne, Victoria, Australia; 16 Department of Data Science and AI, Faculty of IT, Monash University, Melbourne, Victoria, Australia; 17 School of Computing Technologies, RMIT University, Victoria, Australia; 18 Faculty of IT, Monash-Airdoc Research Center, Monash University, Melbourne, Victoria, Australia; Xiangtan Central Hospital, CHINA

## Abstract

Serial cognitive assessment in multiple sclerosis can help identify patients at higher risk of disability progression, but high dimensionality of reaction-time data makes analysis challenging. We developed a method to convert longitudinal reaction-time data into images and applied deep survival modelling to predict disability progression in relapsing-remitting MS (RRMS). In this cohort study, clinical data were obtained from the MSBase registry and cognitive data from the MSReactor computerised cognitive battery between February 2016 and September 2022, with a median follow-up of 3.2 years. The serial reaction-time data from three tasks (psychomotor function [R], attention [G], and working memory [B]) were converted into multicolour images using RGB encoding. We extracted the key features from the images using a convolutional neural network and combined them with clinical variables in a Transformer-based survival model (MS-TranSurv) to predict time to confirmed disability progression. Discrimination, calibration, and accuracy were assessed using the C-index, integrated Brier score (iBS), and time-dependent area under the receiver operating characteristic (tAUROC). Performance was compared with other machine learning models, including Dynamic DeepHit, Recurrent Deep Survival Machines, a Cox proportional hazards model in traditional statistics using clinical variables only, and a version of MS-TransSurv using mean test values. A total of 746 RRMS patients were included. MS-TranSurv showed slightly higher discrimination compared with benchmark models, with a C-index of 0.61 (95% CI 0.54–0.68) and tAUROC of 0.74 (95% CI 0.63–0.85), as well as comparable calibration, with an iBS of 0.24 (95% CI 0.16–0.32). Using individual test-level data provided slightly better performance than using summary measures. Longitudinal cognitive reaction-time data can be used for survival-based prediction of disability progression in RRMS. This framework supports remote cognitive monitoring and may improve risk stratification and clinical trial enrichment, with potential applicability to other high-dimensional digital biomarkers.

## Author summary

People with multiple sclerosis often experience changes in thinking speed, attention, and memory, sometimes years before clear physical disability appears. Digital cognitive tests make it possible to measure these changes repeatedly, including at home, but the large amount of data they produce is difficult to analyse and is often simplified in ways that may lose important information. In this study, we developed a new approach to make better use of these data. We transformed repeated reaction-time measurements from simple cognitive tasks into colour-based images that capture patterns of performance over time. We then used a machine learning method designed to analyse changes over time to estimate the risk of future disability progression. By keeping more detailed information from each test, our approach performed better than methods that rely on summary measures. Our findings show that repeated, low-burden cognitive testing can be used to help identify people with multiple sclerosis who are at higher risk of worsening disability. This approach could support earlier clinical review, improve the selection of participants for clinical trials, and may be adapted for use with other types of digital health data in the future.

## Introduction

Cognitive impairment affects 54–65% of patients with multiple sclerosis (MS) [1]. Processing speed and episodic memory are the most commonly affected cognitive domains [2]. Previous results suggested that the risk of disability progression in patients with relapsing-remitting MS (RRMS) can be predicted by cognitive change trajectories [3,4]. The use of computerised neuropsychological assessment devices (CNADs) makes it simpler and more effective to collect serial cognitive data, including reaction time-based tests [5,6] In addition, CNADs are usually self-administered, therefore, require few or no staff resources and less time to administer, greatly improving the convenience of data collection [7]. However, multiple tasks in a test run and multiple test time points produce large datasets, posing challenges for analysis and interpretation. A common way to analyse these data is to reduce dimensionality by creating summary information, for example, by averaging the reaction time of correct answers over a single testing session [3,8]. However, this method has limitations, as it is susceptible to outliers and does not take into account variations in individual reaction time between different correct responses, informative variation of response times during a test session, and superimposed differential learning effects. Machine-learning (ML) models can map associations that may be too complex to be identified by traditional methods, [9] providing an alternative that can potentially use all the obtained high-volume reaction time data rather than averaging it [10]. However, many existing ML studies in MS rely on binary outcomes defined over fixed time windows, which do not fully capture the time-to-event nature of disability progression or account for censoring. Integrating ML with survival analysis allows longitudinal data to be modelled more appropriately while preserving temporal information.

In this study, we introduce a novel ML model to predict disability progression in RRMS patients by converting high-dimensional reaction time data into multicoloured images and incorporating clinical demographics and characteristics. We extracted key features from reaction images, using a convolutional neural network (CNN), and then used these features to perform Transformer survival models. We collected cognitive data using the MSReactor computerised cognitive battery, which uses reaction time-based tasks to serially monitor cognitive performance in patients with RRMS [11,12]. We hypothesised that incorporating data from each individual test over time improves the performance of the predictive model compared to using the average of all tests. Additionally, we compared the performance of our model with two benchmark ML survival models.

## Methods

### Ethics statement

Clinical data was collected via the MSBase registry (registered with WHO ICTRP, ID ACTRN12605000455662), an international observational cohort study of MS [13]. Ethical approval for the MSBase registry was granted by the Alfred Health Human Research and Ethics Committee and the local ethics committees at participating centres. All participants provided written or verbal consent under local regulations prior to data collection. Demographic and clinical data were collected during routine clinic visits at all study sites via the locally installed iMed or MDS MSBase data entry systems and monitored through a series of procedures to maintain data quality [14]. This study followed the Strengthening the Reporting of Observational Studies in Epidemiology (STROBE) reporting guidelines [15].

De-identified clinical and cognitive data were accessed for research purposes on 15 January 2025. Data security procedures, including data storage, transfer, access control, encryption, and governance arrangements, are described in the supplementary materials (S1 Text). All analyses were conducted on de-identified datasets, and the authors did not have access to any information that could identify individual participants during or after data access.

### Patient population

Patients with RRMS were recruited from 4 tertiary MS clinics in Australia between March 2016 and December 2019. Patients with upper limb, visual, or cognitive deficits preventing the use of touchscreen devices were excluded from the study. An internet-connected tablet or computer was required for optional home-based testing. Ownership of a touchscreen device was not part of the inclusion or exclusion criteria. MSReactor testing was completed during clinic visits approximately every 6 months using iPad tablets provided at each study site, to ensure accessibility and consistency of testing. Participants could also choose to complete optional remote testing using their own tablet or computer, provided that the device had an internet connection to upload results to the MSReactor database. A total of 746 participants with RRMS were included in the final analysis.

### Study design and settings

All participants were enrolled during outpatient visits and provided access to a secure testing website for cognitive assessments. Cognitive data was collected at baseline and approximately every six months during routine clinic appointments. In addition to clinic-based assessments, participants had the option to complete tests at home with an unlimited frequency of testing (monthly testing suggested). Participants completed at least one practice test prior to clinic-based tests and were encouraged to practice before home testing. To ensure sufficient longitudinal cognitive data for model development, participants who completed less than 3 MSReactor tests or were enrolled for less than 180 days were excluded from the study.

The MSReactor computerised cognitive screening battery is a web-based tool designed for self-assessment. It consists of reaction time tasks measuring psychomotor function (simple reaction time [SiRT]), attention (choice reaction time [ChRT]), and working memory (one back [OBK]). Participants respond to stimuli appearing onscreen as quickly as possible, with the system recording response speed in milliseconds and accuracy automatically. Participants continue each task until they achieve at least 30 correct responses. Participants typically completed the reaction time tests in 5 minutes.

### Study endpoint and definitions

The study baseline was defined at the date of the first MSReactor test. The baseline EDSS score was the EDSS score closest to the study baseline obtained within 6 months. The study outcome was a confirmed disability progression event, defined as the confirmed increase in EDSS score of 0.5 or greater for patients with a baseline score greater than 5.5 (or 1.0 or more steps for those with a baseline score between 1.0 and 5.5) and 1.5 or more steps if the baseline score was 0. To detect and confirm disability progression, two subsequent assessments were required, with at least 6 months between assessments [16]. To mitigate the risk of temporary fluctuation being labelled as the baseline EDSS, we excluded EDSS scores obtained within 30 days after a relapse onset date. We defined relapse as having new or recurrent neurologic symptoms lasting for 24 hours or more without fever or infection [17].

### Model development and validation

**Fig 1:**
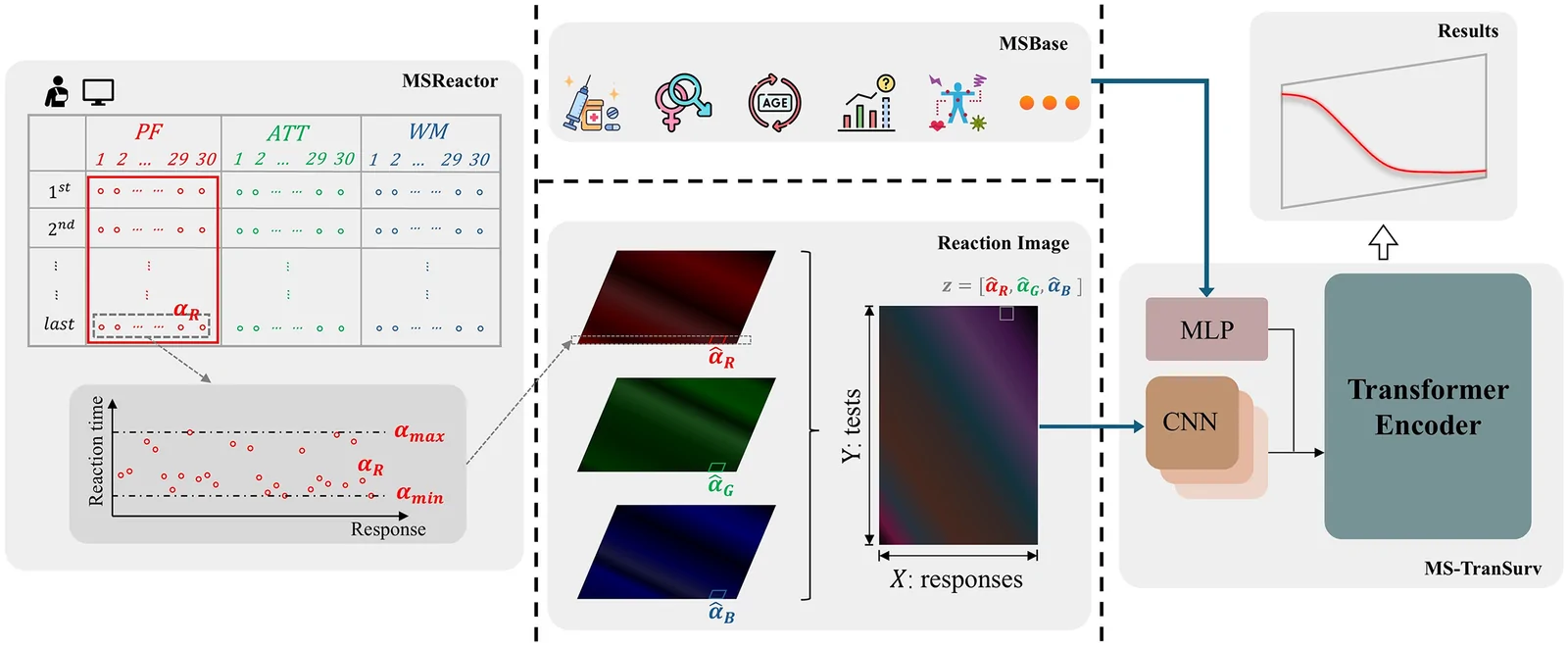
Example of generating a reaction image from individual data and the architecture of MS-TranSurv model. PF = Psychomotor Function (Red), ATT = Attention (Green), WM = Working Memory (Blue), CNN = Convolutional Neural Network, and MLP = Multi-Layer Perceptron.

Our primary goal was to develop a CNN-based Transformer survival (MS-TranSurv) model consisting of a feature extraction module and an encoder module to predict the risk of disability progression. Longitudinal cognitive data were first organised using monthly time points prior to image construction. If multiple MSReactor assessments were completed within the same month, only the last assessment was retained as the representative observation for that month. When no assessment was available for a given month, a variation of the last-observation-carried-forward (LOCF) approach was applied to handle missing longitudinal data. We then converted each patient’s test results (longitudinal set of reaction time test series) into a structured image in order to facilitate the CNN to extract key features from high-dimensional reaction time data (example shown in Fig 1). Each patient has a unique reaction image, where the pixel colour represents the test results encoded using the Red, Green, and Blue (RGB) colour system (a colour system that combines red, green, and blue to produce different colours on an electronic screen [18]. Red, green, and blue correspond to three reaction time tasks (psychomotor function [R], attention [G], and working memory [B]), respectively). The X-axis is the response dimension, with a fixed length of 30, corresponding to patients completing 30 of the three tasks in each test. The Y-axis corresponds to the aligned longitudinal testing sequence, with each horizontal line of pixels corresponding to the results of a single monthly observation (Fig 1). This representation was designed not only to preserve sequential response variability within individual tests, but also to capture potential longitudinal patterns at corresponding response positions across repeated testing sessions, thereby introducing an additional structured dimension beyond a simple one-dimensional temporal sequence. We used the min-max normalisation method to convert the reaction time $\alpha_{i}$ (in milliseconds) for each of the three tasks to a number ${\hat{\alpha }}_{i}$ in the range 0–255, as follows:

${\hat{\alpha }}_{i}=\text{255}\times \frac{\alpha_{i}-\alpha_{min}}{\alpha_{max}-\alpha_{min}},\;i=R,\;G,\;B$,

where the $\alpha_{max}$ and $\alpha_{min}$ represent the maximum and minimum reaction time of each patient for the corresponding task in all tests.

For each cognitive task, individual response-time values were sorted, and the 2nd and 98th percentiles were used as the lower and upper thresholds, respectively. Values below the lower threshold or above the upper threshold were capped at their respective thresholds before normalisation to reduce the influence of extreme values. After obtaining the RGB code, we used *RGB Pillow* (version 10.0.0) to generate the reaction images. Each pixel z in the image consists of a superposition of ${\hat{\alpha }}_{R}$, ${\hat{\alpha }}_{G}$ and ${\hat{\alpha }}_{B}$. The reaction images were first partitioned into image patches according to predefined prediction windows along the longitudinal dimension (S1 Fig). These patches were then processed using a pretrained CNN (developed using the Python package of *PyTorch* [version 2.1]) for feature extraction and dimensionality reduction. The extracted features for each prediction window were subsequently combined with clinical covariates through multiple fully connected layers, forming the extraction module.

The heart of the encoder module was a transformer-based deep survival model [19]. The extracted features were combined and passed to a Transformer encoder via an embedding layer to predict the probability of reaching a confirmed disability progression over time. The Transformer model views each patient as a ‘sentence’, where the combination of positional encoding and feature embedding forms the ‘words’ of that sentence. Each ‘word’ encapsulates the patient’s information at a specific time, allowing the model to efficiently capture intricate temporal patterns within patient data. This unique approach makes the Transformer model a highly effective tool for predicting events over time. The Transformer encoder was trained using a compact architecture due to the limited size of the survival dataset. The CNN-Transformer architecture was adapted from our previously published UniSurv framework, [20] with modifications to accommodate longitudinal cognitive image representations. Detailed architectural specifications, training procedures, hyperparameter settings, and reproducibility information are provided in the supplement (S1 Text). To assess model performance robustly, we employed five-fold cross-validation, randomly partitioning the dataset into training, validation, and test sets with a ratio of 7:1:2 in each iteration.

### Statistical analysis

The performance of the MS-TranSurv model was compared with two benchmark models that support time-varying covariates: the Dynamic DeepHit (DDH) [21] model and the Recurrent Deep Survival Machines (RDSM) [22] model, as well as a Cox proportional hazards (PH) model [23] incorporating only clinical variables as a conventional baseline comparator. DDH and RDSM are multilayer perceptron models that incorporate recurrent neural networks for dynamic analysis of survival data. To determine if the model performance could be improved by using individual test results instead of the mean of all tests, we used the averages and the corresponding standard deviations of all tests to replace the features extracted by CNN (we refer to this model as MS-TranSurv-mean model throughout the rest of the paper). These features were then combined with clinical covariates and passed to the Transformer encoder module. Model performance was assessed by discrimination and calibration. The time-dependent area under the receiver operating characteristic curve (tAUROC) and the Uno survival C-index were used to evaluate the model’s discriminative ability, with values closer to 1 indicating more accurate models [24,25]. The Brier score was applied to estimate the calibration ability, which is the mean squared difference between the observed and the predicted outcome, with a score close to 0 indicating good accuracy and a score close to 1 indicating poor accuracy [26].

All analyses were conducted using either Python version 3.9.17 (Python Software Foundation), or R software version 4.3.2 (R Foundation for Statistical Computing). The level of significance was set at a 2-sided *P* < 0.05.

## Results

### Characteristics of study population

The study population was primarily composed of women (76%), with a mean (SD) age of 42.8 (10.9) years and a median follow-up period of 3.2 years (IQR, [2.1 – 4.6]). Of the 746 included participants, 148 (19%) showed a confirmed disability progression prior to the end of follow-up. Overall, the participants had a median disease duration of 8.5 years (IQR, 4.3 – 13.4), a relatively long treatment exposure duration (median, 5.0 years [IQR, 2.2 – 8.4]), and a low EDSS score at baseline (median, 2.0 [IQR, 1.0 – 3.0]). Characteristics of the included participants are reported in Table 1.

**Table 1:** Clinical Characteristics at baseline^†^.

	Total (n = 746)
Age, yrs	42.8 ± 10.9
Sex	
Female	569 (76)
Male	177 (24)
Disability (EDSS)	2.0 (1.0 – 3.0)
The number of DMT used before baseline^†^	2.0 (1.0 – 3.0)
The number of relapses 1 year before baseline^†^	0.0 (0.0 – 0.0)
The number of relapses 2 years before baseline^†^	0.0 (0.0 – 1.0)
Disease duration, yrs	8.5 (4.3 – 13.4)
Cumulative DMT usage time before baseline, yrs	5.0 (2.2 – 8.4)

Values are median (interquartile range), n (%), or mean ± SD.

† The study baseline was defined at the date of the first MSRactor test.

DMT = Disease-Modifying Therapies; EDSS = Expanded Disability Status Scale.

### Model discrimination

MS-TranSurv model presented the highest discriminative performance with a C-index of 0.61 (95% CI, 0.54-0.68) compared to the DDH model (0.52; [95% CI, 0.40-0.65]) and the RDSM model (0.53; [95% CI, 0.38-0.68]), and the Cox PH model based on clinical variables only (0.53, [95% CI, 0.45-0.61]). The MS-TranSurv model using the individual test data showed a slightly improved C-index relative to the MS-TranSurv-mean model (0.60; 95% CI, 0.54-0.65) using the whole test average, although the 95% CIs overlapped substantially.

**Fig 2:**
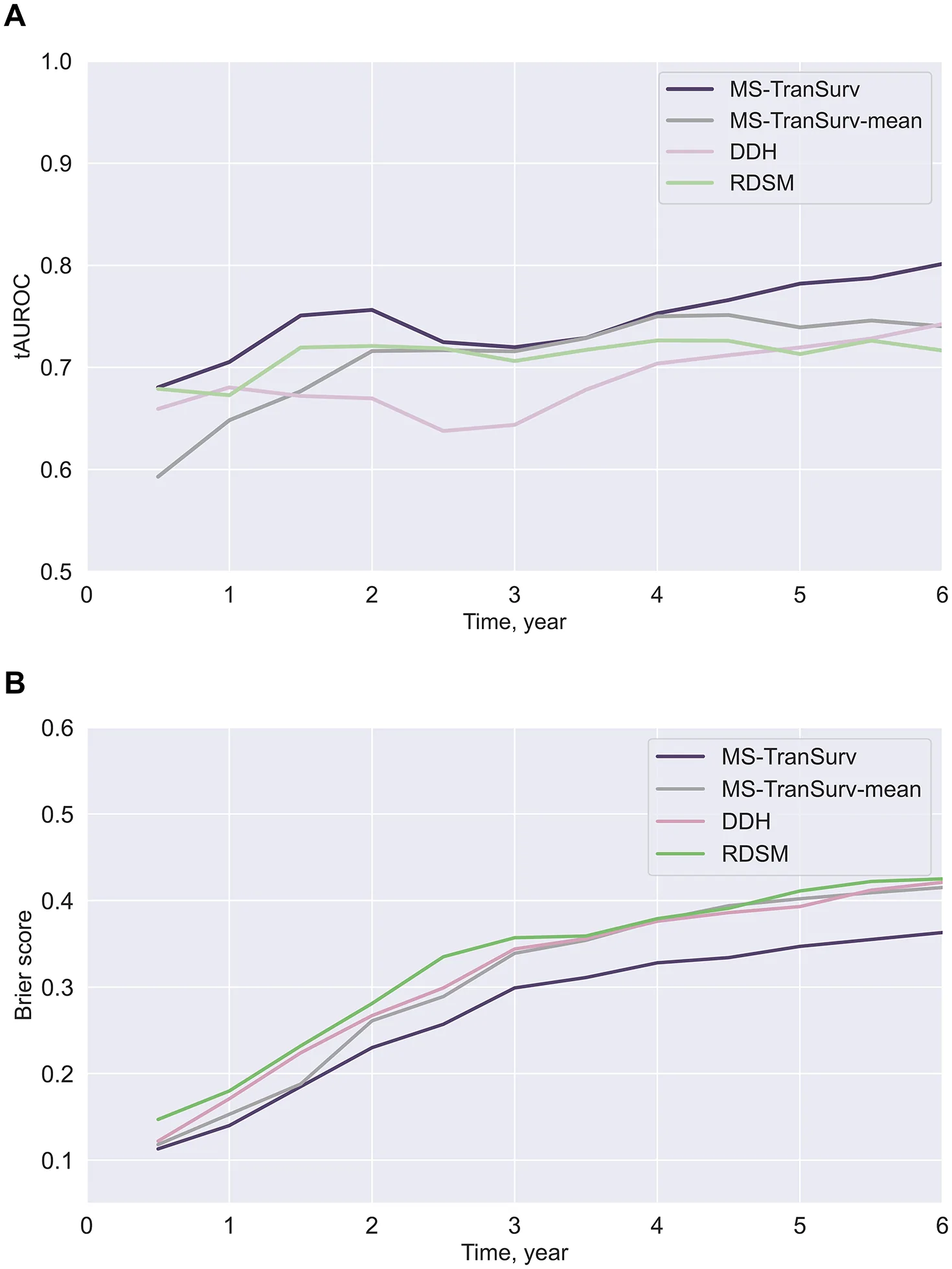
Time-dependent model performance in 6 years. A Time-dependent AUROC tAUROC and B Brier score curves for prediction models calculated at 0.5-year intervals.

MS-TranSurv model achieved an average tAUROC of 0.74 (95% CI, 0.63-0.85), which was numerically higher than the benchmark models (Table 2). We also compared yearly tAUROC across the first five years of follow-up and found that the MS-TranSurv generally showed better discrimination than the other models. The average tAUROC of MS-TranSurv was comparable to that of MS-TranSurv-mean (0.71; 95% CI [0.63-0.79]), with widely overlapped 95% CIs. However, the yearly tAUROC curve for MS-TranSurv was generally above that of MS-TranSurv-mean, except at year 4 when the two models showed similar performance (Fig 2A). The yearly tAUROC values for all four models are reported in the Supplementary (S1 Table).

**Table 2:** Model performances.

	Cox PH	MS-TranSurv	MS-TranSurv-mean	DDH	RDSM
C-index (95% CI)	0.53 (0.45 – 0.61)	0.61 (0.54 – 0.68)	0.60 (0.54 – 0.65)	0.52 (0.40 – 0.65)	0.53 (0.38 – 0.68)
tAUROC (95% CI)	0.60 (0.50 – 0.69)	0.74 (0.63 – 0.85)	0.71 (0.63 – 0.79)	0.67 (0.57 – 0.77)	0.70 (0.63 – 0.76)
Integrated Brier score (95% CI)	0.13 (0.12 – 0.15)	0.24 (0.16 – 0.32)	0.29 (0.14 – 0.43)	0.28 (0.21 – 0.35)	0.29 (0.23 – 0.34)

tAUROC = Time-dependent area under the receiver operating characteristic curve.

### Model calibration

The Cox PH model had the lowest integrated Brier score (iBS) of 0.13 (95% CI, 0.12-0.15), followed by the MS-TranSurv (iBS 0.24; 95% CI, 0.16 - 0.32), DDH (0.28; 95% CI, 0.21-0.35), and RDSM (0.29; 95% CI, 0.23-0.34). MS-TranSurv also showed a lower iBS than MS-TranSurv-mean (0.29; 95% CI, 0.14-0.42), suggesting that incorporating individual test-level longitudinal data may improve calibration compared with using mean cognitive summaries alone. To further assess calibration performance over time, Brier scores were examined at 6-month intervals during follow-up (Fig 2B). MS-TranSurv model generally maintained lower Brier scores than the DDH, RDSM, and MS-TranSurv-mean models across all time points, suggesting more stable calibration performance over time.

**Fig 3:**
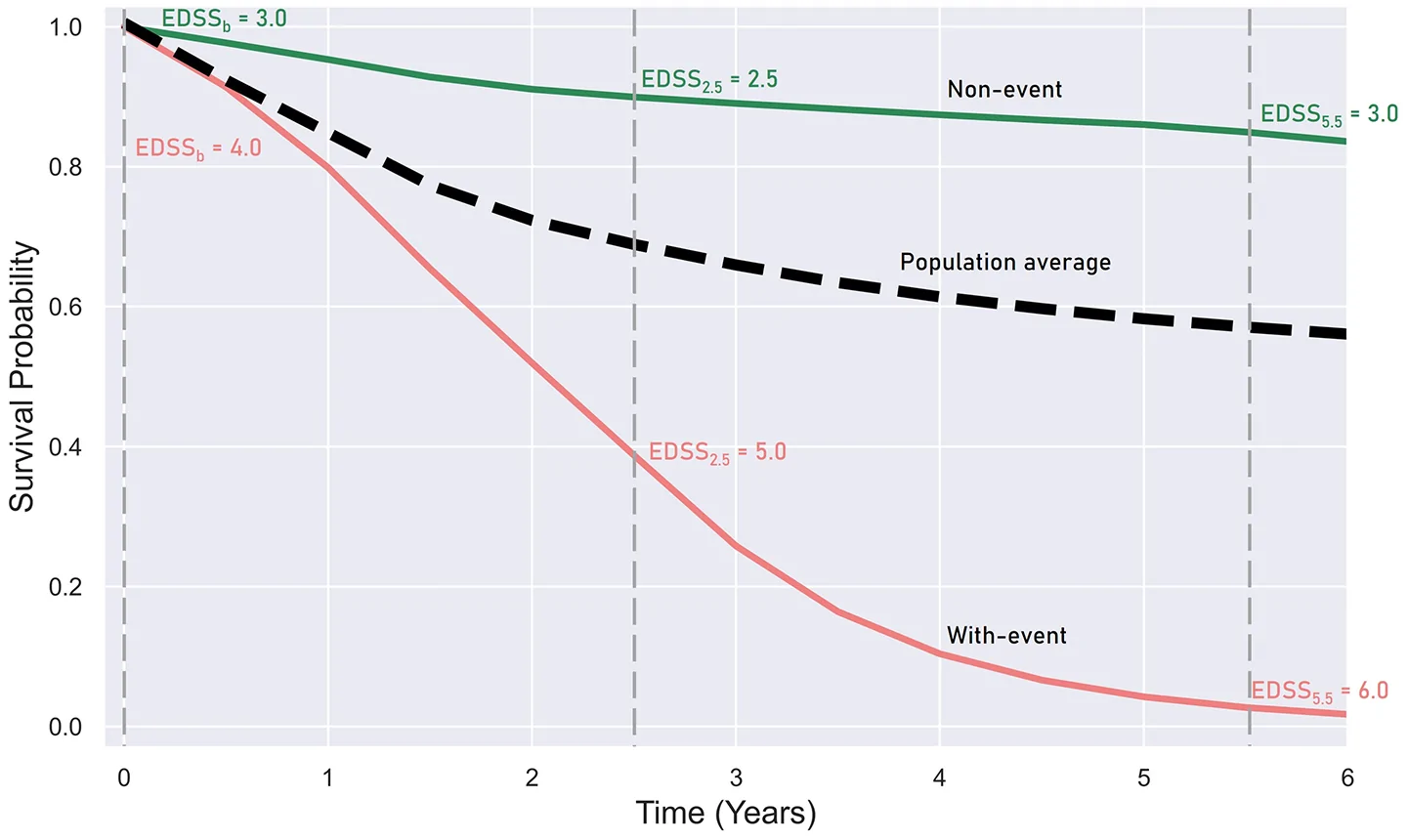
Predicted survival curves for two patients over a six-year period, generated using the MS-TranSurv model. EDSS_b_, EDSS_2.5_, and EDSS_5.5_ were EDSS at the baseline, 2.5 years, and 5.5 years, respectively. The survival curve of with-event (red line) corresponds to a patient who experienced a confirmed disability progression event at 4.58 years. In contrast, the green line depicts the survival curve of a patient who remained event-free and censored at 5.61 years. For reference, population-average survival curves, obtained by averaging the predicted survival probabilities of all individuals in the cohort, are also provided.

Furthermore, we used the MS-TranSurv model to predict survival curves for 2 patients over 6 years (Fig 3) to visually represent what the model can predict. The data utilised for these predictions included one patient who experienced a confirmed disability progression event at 4.58 years, and another patient who remained event-free and was censored at 5.67 years. Additionally, population-averaged survival curves, derived from the average survival probabilities across the entire population over time, were presented for reference.

## Discussion

In this prospective cohort study, we used longitudinal cognitive monitoring data and incorporated common clinical variables to develop a Transformer-based deep-survival model to predict time to disability progression at the individual level in RRMS patients. Compared with the two benchmark models, the MS-TranSurv model demonstrated improved

discrimination and calibration. The MS-TranSurv model using individual cognitive test data could marginally improve the accuracy of predictions compared to the MS-TranSurv-Mean model, which uses the average value of the entire test data.

A growing body of evidence supports the capacity of machine learning (ML) algorithms to extract useful information from images and high-dimensional cognitive data [9,27,28]. Previous studies have demonstrated the advantages of ML algorithms in using MRI information incorporating clinical variables to define biomarkers that predict cognitive changes in MS [28–30]. However, to our knowledge, our CNN-based Transformer survival model is unique in predicting disability progression in MS using longitudinal cognitive data. Our findings extend previous research by providing another way to assess the role of subtle cognitive changes in predicting subsequent disability progression, and provide evidence supporting the feasibility of using repeated, low-burden, automated cognitive data acquisition for predicting disability progression.

Considering the limited sample size, both MS-TranSurv and MS-TranSurv-mean models showed reasonable discrimination and calibration performance. Compared with DDH and RDSM, both models showed numerically better discrimination, while MS-TranSurv also showed a numerically lower iBS than the other deep learning survival models. Interestingly, despite using only clinical variables, the Cox PH model achieved discrimination comparable to DDH and RDSM and showed the lowest iBS, suggesting better average calibration of absolute risk estimates. This difference between discrimination and calibration may reflect that the iBS is sensitive to the accuracy of absolute risk estimates and the overall event rate, whereas the C-index and tAUROC focus on ranking patients by risk. Therefore, a model may achieve a low iBS by estimating average risk well, even if it has limited ability to separate higher-risk from lower-risk patients [31]. These findings indicate that simply incorporating longitudinal cognitive data into a survival model may not automatically improve predictive performance. Instead, how high-dimensional longitudinal cognitive signals are represented and processed appears to be important. The better discrimination observed with MS-TranSurv suggests that the proposed framework may extract informative longitudinal cognitive features that were not fully captured by the other benchmark approaches. Moreover, MS-TranSurv showed a slightly higher average tAUROC point estimate than benchmark models, suggesting a potential benefit of incorporating individual-level test data. However, the substantial overlap in their 95% CIs, likely due to the limited sample size, indicates that this improvement might be marginal. In particular, the identical performance of the two models at year 4, could also be attributed to a large number of patients being censored during that period.

In addition, the longitudinal Brier score trajectories suggest that calibration deteriorated more gradually over time for MS-TranSurv than for the other deep learning models, although confidence intervals remained overlapping. The Cox PH model demonstrated the strongest overall calibration performance (S1 Table), which may reflect the stability of simpler assumption-based statistical models in relatively limited datasets [20,32]. However, the superior discrimination achieved by MS-TranSurv suggests that effective extraction of longitudinal cognitive features may provide additional prognostic information beyond conventional clinical variables alone. As our cohort is still ongoing and the sample size is increasing, we anticipate further improvement in the accuracy of the model.

CNN has proven highly effective in recognising patterns within image data and handling complex image recognition tasks [33,34]. It also offers benefits in dimensionality reduction by learning low-dimensional representations of high-dimensional data and extracting hierarchical features while retaining essential information and enhancing interpretability [35,36]. Leveraging these advantages of CNN in processing high-dimensional data, we generated images based on each participant’s cognitive test data and then input them into the CNN to extract features for the encoder module. This approach yielded superior results compared to directly averaging all test results.

Reaction-time data may include fluctuations related to fatigue, learning effects, or occasional extreme responses. In this study, extreme values were capped using the task-specific 2nd and 98th percentile thresholds before min–max normalisation, reducing the influence of outliers while retaining potentially informative variation in response-time patterns. However, this approach may not fully remove the influence of measurement variability or extreme responses. Importantly, the image-based representation and CNN feature extraction were designed to learn patterns across repeated responses rather than depend on single response-time values. In addition, the availability of multiple repeated responses within each test session further reduces the influence of extreme values on feature extraction and prediction.

The overall framework of converting high-dimensional longitudinal signals into images and using a CNN-Transformer pipeline is not limited to reaction-time data. This approach can be applied to other complex data streams such as smartphone-derived digital biomarkers, gait sensor outputs, or high-dimensional proteomic and immunologic time-series. This suggests potential applicability in other MS research settings where detailed longitudinal patterns are informative but difficult to model with traditional methods. Using MSReactor, each test session generated approximately 90 variables (30 reaction times across each of three tasks). However, other digital performance tests can generate thousands of data points within seconds (for example, drawing tasks on touchscreens), which are typically heavily summarised before analysis [37]. Our approach could instead represent these high-dimensional data as multicoloured images, allowing the model to capture information and hidden features that would otherwise be lost when the data are summarised.

More broadly, this framework aligns with the emerging concept of digital phenotyping, in which high-frequency behavioural and cognitive data are used to characterise individual-level patterns and derive digital biomarkers for disease monitoring and prediction. Recent work has highlighted the potential of small-data machine learning approaches to capture meaningful behavioural patterns even in settings with limited sample sizes [38]. In this context, repeated longitudinal cognitive testing may provide individualised digital behavioural features that are difficult to capture using conventional clinical variables alone and may ultimately support personalised monitoring and prediction of disease progression in MS.

There are also clear methodological advantages over conventional ML survival models. The CNN captures important within-test patterns that are lost when using mean values, and the Transformer encoder is well-suited for learning long-range dependencies over irregularly collected cognitive data. This likely explains the consistently better discrimination and calibration observed with MS-TranSurv and highlights the value of preserving temporal structure rather than merely relying on summary statistics [39].

Importantly, MS-TranSurv may also have practical clinical relevance. Serial digital cognitive testing is low cost, can be completed outside routine clinic visits, and requires minimal clinical staff time. Previous studies have also demonstrated good long-term acceptability of smart-device monitoring among people living with MS [40,41]. By capturing longitudinal cognitive change, MS-TranSurv may help identify early signals of disability progression and support timely clinical review [42]. Individualised progression risk estimates may also have value in clinical trial design by helping identify patients at higher progression risk, thereby improving trial efficiency and potentially reducing required sample sizes. However, the overall discriminative performance remained moderate and external validation has not yet been performed. Therefore, the current findings should primarily be interpreted as supporting the feasibility of longitudinal cognitive deep-survival modelling rather than a fully clinically validated prediction tool at the current stage. Further validation in larger and independent cohorts will be necessary before clinical implementation [43].

The strategies employed in the MS-TranSurv model for handling complex, high-dimensional data could potentially be adapted for application in other domains as well. For example, converting high-dimensional flow cytometry and serum proteomics data into images and then using CNN for dimensionality reduction and feature extraction to predict disease progression in MS [44].

### Limitations

The model did not include other variables that could further improve the prediction of disability progression, such as comorbidities, BMI, and quality of life, as they are not yet routinely collected in MSBase. Previous studies have demonstrated that baseline MRI features, especially brain atrophy, are strong predictors of future cognitive decline and disability progression [45]. However, as MRI information collection was optional in MSBase, with more than 60% of MRI data missing, MRI data was unavailable for inclusion. Follow-up duration is another limitation. The median follow-up of 3.2 years may be relatively short for detecting disability progression in MS. Although the survival modelling framework accounts for right-censored observations and varying follow-up times, limited follow-up may reduce the number of observed progression events and restrict the ability to capture longer-term progression patterns. These factors may affect model training and estimation of longer-term progression risk. In addition, the lack of external validation increases the likelihood of overfitting and may reduce model performance when applied to data from different cohorts. Ongoing data collection and future external validation in cohorts with longer follow-up will therefore be important to assess the stability, calibration, and generalisability of MS-TranSurv.

Furthermore, the current image transformation approach was based on a relatively simple min-max normalisation and RGB encoding strategy and did not systematically investigate how different time-series-to-image transformation methods may influence preservation of temporal correlations or feature interpretability. More mathematically grounded approaches, such as Gramian Angular Field or Markov Transition Field representations, [46,47] may represent valuable directions for future work. Also, home/hospital-based testing frequency varied substantially across participants. Although monthly discretisation and a variation of the LOCF approach were used to standardise irregular longitudinal observations, the model may still have represented patients with more frequent or regular testing more effectively than those with sparse assessments. MSReactor assessments were also completed during routine clinic visits approximately every 6 months, providing a consistent source of cognitive data and reducing reliance on variable home-testing frequency alone. Future studies incorporating more flexible time-aware architectures or optimising the temporal resolution of the model relative to the underlying data structure may further improve prediction performance and survival trajectory estimation.

A further limitation is potential selection bias related to the requirements for digital task completion. MSReactor was developed as a digital cognitive monitoring tool for people with RRMS who can complete repeated device-based cognitive assessments. Participants were required to be able to use a touchscreen device and complete at least three MSReactor assessments, and patients with upper limb, visual, or cognitive deficits that prevented reliable task completion were excluded. These criteria were necessary to ensure reliable task completion and sufficient longitudinal data for model development. However, this may have resulted in the under-representation of patients with greater barriers to digital testing, limiting the generalisability of MS-TranSurv to the broader MS population. Despite this limitation, the findings support the potential of smartphone-based cognitive monitoring as a practical approach for capturing longitudinal cognitive changes that may provide early signals of future disability progression. Future external validation in broader clinical cohorts and further development of accessible digital assessment platforms will be important before wider clinical implementation.

## Conclusion

In this cohort study, we developed and validated the MS-TranSurv model, a risk assessment tool incorporating cognitive data to predict disability progression in RRMS. Compared with benchmark models, MS-TranSurv demonstrated improved discrimination and calibration performance, supporting the feasibility of incorporating longitudinal cognitive data into deep-survival modelling frameworks for disability progression prediction in MS. The results could help stratify potential trial participants to select subgroups with higher disability progression risk for clinical trials and ultimately prove useful for individual disease and therapeutic response monitoring. Our novel method is potentially applicable to digital and other datasets with much higher dimensionality than MSReactor, and could discover hidden data features with prognostic significance in them.

### Data Access, Responsibility, and Analysis

C. Zhu and X. Zhang had full access to all of the data in the study and took responsibility for the integrity of the data and the accuracy of the data analysis.

## Supporting information

S1 Text MS–TranSurv Architecture, Training Procedures, and Prediction. This file includes additional details on the MS-TranSurv architecture, longitudinal data preprocessing, image construction, model training procedures, prediction framework, survival loss function, data security procedures, and supplementary model performance results.
(DOCX)

S1 Fig Overview of the MS-TranSurv architecture. Reaction image patches and patient-level clinical information are processed through separate feature extraction modules. The extracted latent features are then fused and passed into a Transformer encoder to estimate survival probabilities over time.
(DOCX)

S1 Table Model performance with yearly tAUROC and Brier scores. This table reports C-index, integrated Brier score, average tAUROC, yearly tAUROC, and yearly Brier scores for the Cox PH, MS-TranSurv, MS-TranSurv-mean, DDH, and RDSM models.
(DOCX)
