# Supplementary material for: Strategies for integrating artificial intelligence and cognitive assessment to predict disability progression in relapsing-remitting multiple sclerosis: A model development study
Source: PLOS Digit Health. 2026 Sep 25;5(9):e0001722. doi: 10.1371/journal.pdig.0001722 (PMC13614581; doi:10.1371/journal.pdig.0001722)
Supplement: Supplementary file 1 [file pdig.0001722.s001.docx]

**Appendix: MS–TranSurv Architecture, Training Procedures, and Prediction Framework**

**Flow of information through the MS-TranSurv network:** We employed a deep learning model, MS-TranSurv, to predict individual survival probabilities for patients. As the overview shown in eFigure 1, MS-TranSurv consists of three main components: a dynamic extraction module, a static extraction module, and a survival encoder.

Longitudinal cognitive observations were first organised into monthly time points. If multiple MSReactor assessments were completed within the same month, only the last assessment was retained as the representative observation for that month. Meanwhile, only observations up to the event/censoring time were included for uncensored/censored participants. Missing monthly observations were handled using a variation of the last-observation-carried-forward (LOCF) approach, and the shorter longitudinal trajectories were padded using the same LOCF strategy after alignment to the predefined prediction timeline to enable construction of uniformly sized image representations. Min-max normalisation was performed as part of the image-construction process using the longitudinal cognitive observations included in the analysed trajectory. This normalisation procedure was used solely for image construction and standardisation of within-subject variability. Outcome information and event labels were not used during image construction. The longitudinal cognitive observations were subsequently transformed into reaction images and partitioned into image patches along the longitudinal dimension according to predefined prediction window ($T_{w}=8$ months). Each patch therefore contained information from its corresponding prediction window and was processed independently by the dynamic extraction module.

The dynamic extraction module consisted of a pre-trained MobileNetV2^1^ backbone, which was used to extract time-varying embedded features from the image patches. Simultaneously, patient-level clinical variables were processed by the static extraction module, which consisted of a multilayer perceptron (MLP), to generate time-invariant features. The extracted time-varying and time-invariant features were concatenated and sequentially passed to the survival encoder, which was implemented using a Transformer architecture. The model estimates a survival trajectory rather than generating a single prediction based on the entire follow-up period. Survival probabilities are produced sequentially at each time point and jointly optimised through the ordinal survival loss function. Because observations within the same prediction window contribute to a shared latent representation, neighbouring time points are linked through a common feature representation, allowing the model to estimate a smooth and temporally coherent survival trajectory. To prevent future observations from being used in earlier predictions, the Transformer encoder employs a causal masking mechanism within the self-attention layer.^2^ Consequently, predictions at a given time point can only attend to information available up to that time point.

This overall CNN-Transformer framework was adapted and extended from our previously published UniSurv^3^ architecture, with modifications to accommodate longitudinal cognitive image representations and the use of an ordinal survival regression loss for survival trajectory estimation. In addition, a lightweight MobileNetV2 backbone and a downsized Transformer encoder were employed to reduce model complexity and mitigate overfitting risk in the relatively limited dataset. Detailed architectural specifications, feature dimensions, and training procedures of the underlying UniSurv framework have been reported previously.^3^ In the present study, hyperparameter spaces are summarised in the following, with the bolded optimal parameters:

- Prediction windows $T_{w}$: $\{4, 6,\boldsymbol{8},12, 24\}$
- Epochs: $\boldsymbol{200}$
- Batch size: $\{8, \boldsymbol{16}, 32\}$
- Transformer - number of heads: $\{4, \boldsymbol{8}, 16\}$
- Transformer - embedding dimension: $\{256, \boldsymbol{512}\}$
- Transformer - number of attention layers: $\{2, 3, \boldsymbol{4}, 5\}$
- Transformer - dropout rate: {0.0, **0.1**, 0.2, 0.3}
- Adam optimizer with learning rate: $\{1e-3, \boldsymbol{1}\boldsymbol{e-4}\}$

The hyperparameters were selected based on validation performance during five-fold cross-validation. All experiments were conducted using a fixed random seed ($=42$) to improve reproducibility.

**Specific survival loss used by the model:** We employed a nonparametric survival analysis in this work. Given the potential calibration issues associated with survival loss functions used in UniSurv, we adopted an ordinal survival regression loss^4^ to optimize survival probabilities at each time point. This approach predicts the complement of the hazard function, which is then multiplied sequentially to produce the final survival trajectory while preserving the monotonic decrease of survival probabilities over time.

**Details on data storage, data transfer, access control, encryption, and governance arrangements:** De-identified clinical and cognitive data were accessed for research purposes on 15 January 2025. Identifying information was stored separately from clinical and cognitive test data at all times, and the two datasets could not be linked without authorised access. Data were encrypted using 256-bit encryption and protected during transmission using Secure Sockets Layer technology. Access to de-identified clinical data was restricted to approved investigators at each study site and limited to data from their own site. The encryption key required to link records to individual cognitive information was restricted to the study principal investigators. The MSReactor platform is owned by Monash University, and de-identified datasets extracted from the database were stored on secure Monash infrastructure and accessed only by approved investigators. Data access and use were conducted under the relevant study governance and approval arrangements.

**References:**

1. Sandler M, Howard A, Zhu M, Zhmoginov A, Chen LC. MobileNetV2: Inverted residuals and linear bottlenecks. In: *Proceedings of the IEEE Conference on Computer Vision and Pattern Recognition (CVPR)*. IEEE; 2018:4510-4520.
2. Vaswani A, Shazeer N, Parmar N, Uszkoreit J, Jones L, Gomez AN, Kaiser Ł, Polosukhin I. Attention is all you need. In: *Advances in Neural Information Processing Systems 30 (NIPS 2017)*. Curran Associates, Inc.; 2017:5998-6008.
3. Zhang X, Mehta D, Hu Y, Zhu C, Darby D, Yu Z, Merlo D, et al. Adaptive transformer modelling of density function for nonparametric survival analysis. *Mach Learn*. 2025;114(2):31.
4. Hu, S., Fridgeirsson, E., van Wingen, G., & Welling, M. (2021, May). Transformer–based deep survival analysis. In *Survival Prediction–Algorithms, Challenges and Applications* (pp. 132–148). PMLR.
