# Supplementary material for: Strategies for integrating artificial intelligence and cognitive assessment to predict disability progression in relapsing-remitting multiple sclerosis: A model development study
Source: PLOS Digit Health. 2026 Sep 25;5(9):e0001722. doi: 10.1371/journal.pdig.0001722 (PMC13614581; doi:10.1371/journal.pdig.0001722)
Supplement: Supplementary file 2 [file pdig.0001722.s002.docx]

**Fig. Overview of the MS–TranSurv architecture.**

The reaction image patches and meta–information for individual patients are processed through separate modules for feature extraction. The latent features are then fused and fed into a Transformer Encoder, which computes attention across different time points. The output from the Transformer is fed into an MLP, which generates the final survival curve through a sigmoid layer, optimized using a specific survival loss function.
