# Supplementary material for: Strategies for integrating artificial intelligence and cognitive assessment to predict disability progression in relapsing-remitting multiple sclerosis: A model development study
Source: PLOS Digit Health. 2026 Sep 25;5(9):e0001722. doi: 10.1371/journal.pdig.0001722 (PMC13614581; doi:10.1371/journal.pdig.0001722)
Supplement: Supplementary file 3 [file pdig.0001722.s003.docx]

| **Table. Model Performances with yearly tAUROC.** | | | | | | | | |  |  |  |  |  |
| --- | --- | --- | --- | --- | --- | --- | --- | --- | --- | --- | --- | --- | --- |
|  | **C-index** | **Integrated Brier score** | **tAUROC** | **tAUROC-1 year** | **tAUROC-2 year** | **tAUROC-3 year** | **tAUROC-4 year** | **tAUROC-5 year** | **Brier score-1 year** | **Brier score-2 year** | **Brier score-3 year** | **Brier score-4 year** | **Brier score-5 year** |
| **Cox PH** | 0.527 (0.446 – 0.607) | 0.132 (0.116 – 0.149) | 0.597 (0.501 – 0.693) | 0.618 (0.504 – 0.732) | 0.613 (0.536 – 0.689) | 0.617 (0.526 – 0.708) | 0.618 (0.537 – 0.700) | 0.590 (0.509 – 0.671) | 0.071 (0.053 – 0.089) | 0.117 (0.099 – 0.135) | 0.152 (0.140 – 0.165) | 0.153 (0.135 – 0.172) | 0.163 (0.140 – 0.185) |
| **MS-TranSurv (95% CI)** | 0.610 (0.539 – 0.681) | 0.241 (0.162 – 0.320) | 0.743 (0.632 – 0.853) | 0.745 (0.640 – 0.851) | 0.733 (0.648 – 0.819) | 0.723 (0.670 – 0.775) | 0.758 (0.664 – 0.851) | 0.790 (0.679 – 0.901) | 0.140 (0.096 – 0.192) | 0.230 (0.168 – 0.301) | 0.299 (0.243 – 0.368) | 0.328 (0.267 – 0.405) | 0.347 (0.278 – 0.421) |
| **MS-TranSurv-mean (95% CI)** | 0.598 (0.543 – 0.652) | 0.285 (0.143 – 0.428) | 0.708 (0.626 – 0.790) | 0.660 (0.586 – 0.743) | 0.714 (0.585 – 0.842) | 0.732 (0.587 – 0.877) | 0.785 (0.694 – 0.876) | 0.745 (0.656 – 0.834) | 0.153 (0.096 – 0.232) | 0.261 (0.194 – 0.328) | 0.339 (0.274 – 0.426) | 0.377 (0.313 – 0.447) | 0.402 (0.362 – 0.483) |
| **DDH (95% CI)** | 0.521 (0.396 – 0.647) | 0.278 (0.208 – 0.347) | 0.672 (0.572 – 0.773) | 0.672 (0.493 – 0.850) | 0.647 (0.541 – 0.752) | 0.675 (0.519 – 0.831) | 0.713 (0.532 – 0.895) | 0.723 (0.575 – 0.872) | 0.171 (0.113 – 0.218) | 0.267 (0.199 – 0.328) | 0.344 (0.258 – 0.439) | 0.376 (0.296 – 0.454) | 0.393 (0.307 – 0.472) |
| **RDSM (95% CI)** | 0.526 (0.375 – 0.678) | 0.288 (0.232 – 0.343) | 0.698 (0.632 – 0.763) | 0.708 (0.604 – 0.812) | 0.724 (0.665 – 0.783) | 0.705 (0.543 – 0.867) | 0.734 (0.625 – 0.844) | 0.731 (0.611 – 0.851) | 0.180 (0.135 – 0.226) | 0.281 (0.214 – 0.342) | 0.357 (0.294 – 0.442) | 0.379 (0.299 – 0.466) | 0.411 (0.353 – 0.495) |
| tAUROC = Time-dependent area under the receiver operating characteristic curve. | | | | | | | | | | | | | |
